# Different Risk Target Volumes for Nasopharyngeal Carcinoma Treated with Simultaneous Integrated Boost Intensity-Modulated Radiotherapy

**DOI:** 10.7150/jca.45767

**Published:** 2020-07-06

**Authors:** Fangzheng Wang, Chuner Jiang, Lei Wang, Fengqin Yan, Yongfeng Piao, Zhimin Ye, Min Xu, Jiping Liu, Zhenfu Fu, Yangming Jiang

**Affiliations:** 1 Department of Radiation Oncology, Cancer Hospital of the University of Chinese Academy of Sciences, Zhejiang Hangzhou 310022, People's Republic of China.; 2 Institute of Cancer and Basic Medicine (ICBM), Chinese Academy of Sciences, Zhejiang Hangzhou 310022, People's Republic of China.; 3 Department of Radiation Oncology, Zhejiang Cancer Hospital, Zhejiang Hangzhou 310022, People's Republic of China.; 4 Key Laboratory of Radiation Oncology of Zhejiang Province, Zhejiang Hangzhou 310022, People's Republic of China.; 5 Department of Breast Tumor Surgery, Cancer Hospital of the University of Chinese Academy of Sciences, Zhejiang Hangzhou 310022, People's Republic of China.; 6 Department of Breast Tumor Surgery, Zhejiang Cancer Hospital, Zhejiang Hangzhou 310022, People's Republic of China.; 7 Department of Digital Earth, Institute of Remote Sensing and Digital Earth, CAS, Beijing, 100101, People's Republic of China.

**Keywords:** nasopharyngeal carcinoma, IMRT, target volume, dose, long-term survival

## Abstract

**Background and Objectives**: Although intensity-modulated radiotherapy (IMRT) provides promising survival advantages and fewer late complications in patients with nasopharyngeal cancer (NPC), appropriated target volumes and prescribed doses are still being explored. This study aimed to propose different risk target volumes and corresponding prescribed doses in our center and to evaluate the physical basis and efficacy of this protocol based on the long-term survival of NPC patients.

**Methods and Materials:** We retrospectively assessed patients with histology-proven non-metastatic NPC treated with definitive IMRT using our protocol of different risk target volumes and corresponding prescribed doses based on the orderly stepwise pattern of tumor spread. We described the delineation for different risk target volumes and the design of IMRT planning for an NPC case. Additionally, we compared the dosimetric distributions between the China protocol and our protocol through two NPC cases. The patterns of failure and locoregional control were the primary endpoints. All survival outcomes were calculated using the Kaplan-Meier method.

**Results:** From January 2013 to December 2014, a total of 335 patients were treated; the median follow-up for patients who survived was 70 months. All patients completed IMRT using our protocol. Twenty-five patients developed locoregional recurrence, and all recurrences occurred within the high-dose target volumes. The rates of locoregional recurrence-free survival, distant metastasis-free survival, progression-free survival, and overall survival at 5 years were 92.2%, 92.1%, 85.9%, and 86.3%, respectively. The biological effective doses of the prescribed doses in our protocol were similar to those of the China and 0615 protocols. Moreover, our protocol offered a reduction in D1 and D2 in the primary gross tumor volume (GTV), while V30 and V40 in normal tissues were lower.

**Conclusion:** Our protocol of different risk target volume delineations and corresponding prescribed doses based on the stepwise pattern of tumor spread resulted in favorable locoregional control with no relapse outside the GTV.

## Introduction

Nasopharyngeal cancer (NPC), a unique malignancy of the head and neck, is endemic to southeast Asia, northern Africa, and middle Europe where the acute incidence is 15-50 cases per 100,000 annually 1. The latest GLOBOCAN data 2 in 2018 reported that 129,097 patients were newly diagnosed with NPC worldwide. Of these patients, 47.7% were in China.

Due to the high sensitivity to radiation and the complicated anatomical structure of the nasopharynx, radiation therapy (RT) is regarded as the mainstay of treatment for NPC. Previously, conventional two-dimensional RT was a common technique for NPC. In early reports, the 5-year survival rate of NPC after two-dimensional RT was 67-76% 3-7. However, late complications, including carotid stenosis, optic neuropathy, and brain necrosis, reduced the quality of life in long-term survivors 8-10.

Compared with two-dimensional RT, intensity-modulated radiation therapy (IMRT) could improve the dose covering the clinical target volume (CTV) in three dimensions while protecting the normal tissues around the CTV 11. ]. Moreover, IMRT provides favorable local control and survival outcomes in NPC 12-18. In contrast to non-IMRT techniques, IMRT improves cancer-specific survival for head and neck cancer according to the Surveillance, Epidemiology, and End Results (SEER) databases, especially in NPC patients 19. IMRT can be implemented using a sequential (SEQ) or simultaneous integrated boost (SIB) protocol. SEQ IMRT uses a two-phase shrinkage technique, while SIB IMRT delivers different dose levels to different target volumes. Compared with SEQ IMRT, SIB IMRT yields more satisfactory dosimetric outcomes for nearby critical organs and is regarded as the standard therapy for NPC 20-22. In addition, whole-field SIB IMRT provides more dosimetric benefits for the larynx and fewer set-up errors than junction IMRT with a conventional RT field 23.

Currently, due to the introduction of Magnetic Resonance (MR) and induction chemotherapy (IC), IMRT has significantly improved the survival rate of NPC to 80-85% 12-14. A recent study reported that the locoregional relapse-free survival (LRRFS), distant metastasis-free survival (DMFS), and disease-specific survival (DSS) of 865 NPC patients in 2001-2008 were 92%, 83.4%, and 78.6%, respectively, 10 years after IMRT 24. However, insufficient RT dose is still one of the most important factors affecting the therapeutic effect. In many previous protocols 12-14, 16-18, bone mark and wide margins from the GTV of the nasopharynx and cervical lymph nodes were used to encompass the high-dose area. These experiences came from conventional two-dimensional RT techniques. Although IMRT provides encouraging local control and survival outcomes, normal tissues are also exposed to unnecessarily high doses, and some late complications such as hearing loss, carotid stenosis, optic neuropathy, and brain necrosis occur in long-term survivors 8-10, 25. Ng and colleagues 26 evaluated the effect of dosimetric inadequacy in target volumes on the local control of NPC; the results indicated that if the volume in the gross tumor volume (GTV) below 66.5 Gy was more than 3 cc, the 5-year rate of local failure-free survival dropped to 54%. Both target volumes and prescribed doses are vital for IMRT in NPC. Recently, a global questionnaire study proposed a CTV design scheme for NPC, i.e., the principle of “5 + 5 mm expansion.” Based on the GTV, the high-risk CTV is formed by expanding 5 mm, and then the low-risk CTV is formed by a further expansion of 5 mm 27. However, there are no available data regarding the expansion of NPC tumors and no radical dose for the GTV. If the CTV is obtained by uniform expansion from the GTV, excessive normal tissue may be irradiated. Therefore, reduced target volumes have been used to apply IMRT in NPC 15-17. However, optimal target volumes and doses of IMRT in NPC remain unclear due to a lack of available data on the extent of the invasion of the NPC tumor.

Considering the above situation, we designed a protocol for different risk target volumes and corresponding prescribed doses in SIB IMRT based on the characteristics of the gradual spread of the tumor. The main objectives were to introduce our protocol and investigate the long-term results and adverse events in patients with NPC treated with our protocol.

## Materials and Methods

### Patients

Between January 2013 and December 2014, 355 patients hospitalized at the Department of Radiation Oncology at Zhejiang Cancer Hospital were retrospectively reviewed. Eligible patients met the following criteria: (i) biopsy-proven NPC, (ii) no metastasis occurred, (iii) received SIB IMRT using our protocol, (iv) completion of treatment, and (v) no previous anti-cancer treatment. Patients who did not complete definitive IMRT, those without metastasis at diagnosis, or those who had received previous anti-cancer treatment were excluded. This retrospective study was approved by the Medical Ethics Committee and the Institutional Review Board of Zhejiang Cancer Hospital. All treatment protocols in this study were carried out in accordance with the National Comprehensive Cancer Network guidelines. Due to the retrospective design of the study, the committee confirmed that informed consent was not required.

### Pretreatment evaluations

Pretreatment evaluations included the following: detailed medical histories, evaluation of performance status, and careful physical examinations. Magnetic resonance imaging (MRI) of the nasopharynx and nasopharyngoscopies was performed. Chest computed tomography (CT), bone scans, abdominal ultrasound, and hematology and biochemistry tests were conducted 1 week before treatment. Positron emission tomography scans and abdominal CT scans were performed as clinically indicated. The 2010 American Joint Committee on Cancer staging system and World Health Organization classification were recommended for use in this study.

## SIB IMRT

### Body position fixation and scanning requirements

All patients receiving SIB IMRT were immobilized in the supine position using thermoplastic masks. CT scans with intravenous contrast using 2.5-mm slices from the head to a level 2 cm below the sternoclavicular joints were performed for planning. Intravenous contrast was administered at approximately 1-2 mL/kg/s.

### Names and relationship of different risk target volumes

**Figure 1:**
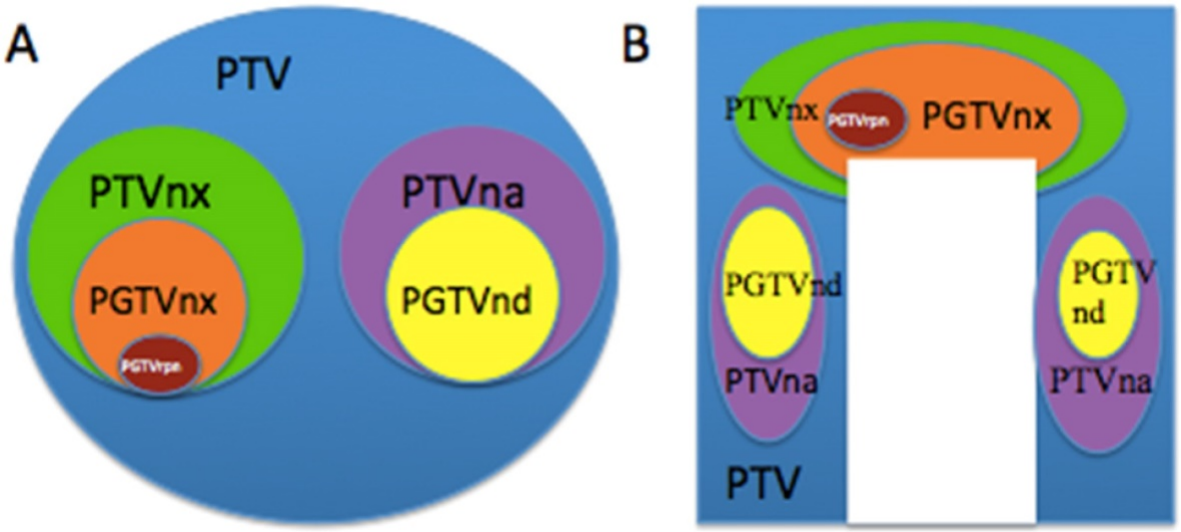
** The different risk target volumes.** (**A**) Cross section; (**B**) coronary position. Red: PGTVrpn; orange: PGTVnx; yellow: PGTVnd; green: PTVnx; pink: PTVna; blue: PTV. Abbreviations: PGTVrpn: planning target volume of GTV in retropharyngeal lymph nodes; PGTVnx: planning target volume of nasopharyngeal GTV; PGTVnd: planning target volume of GTV in cervical lymph nodes; PTVnx: planning target volume of nasopharynx; PTVna:planning target volune of neck area; PTV: planning target volume.

Based on the orderly stepwise pattern of tumor spread, we designed three or four risk target volumes in the pathway of NPC tumor invasion including the peripheral (Figure 1A), upper, and lower structures (Figure 1B). The corresponding target volumes included high-risk planning target volume of nasopharyneal GTV (PGTVnx) or planning target volume of GTV in cervical lymph nodes (PGTVnd) encompassing GTV of nasopharynx (GTVnx) or GTV of cervical lymph nodes (GTVnd), intermediate-risk planning target volume of nasopharyx (PTVnx) or planning target volume of neck area (PTVna) encompassing CTV of nasopharynx (CTVnx) or CTV of neck area (CTVna), and low-risk planning target volume (PTV) encompassing CTV. If the retropharyngeal lymph nodes (RLNs) were large or resistance to IC, we add a higher risk target volume for PTV of GTV in retropharyngeal lymph nodes (PGTVrpn) encompassing GTV of retropharyngeal lymph nodes (GTVrpn). The relationship of the three to four risk tumor volumes such as PGTVnx (PGTVrpn), PGTVnd, PTVnx, PTVna, and PTV is shown in Figure 1.

### Definitions of the different risk target volumes

According to the orderly stepwise pattern of tumor spread, we designed the different risk target volumes as follows. GTV referred to the macroscopic tumor extent found in clinical and imaging baseline examinations or before treatment. The primary tumor extent including the metastatic retropharyngeal lymph nodes (RLNs) was named the GTVnx, and the metastatic lymph nodes of the neck were named the GTVnd. If the RLNs were large and resistant to IC, we add a GTVrpn into the GTVnx. Based on the above for each GTV, we automatically expanded the margin by an additional 1-3 mm in three dimensions to get the corresponding PTVs, including PGTVnx, PGTVnd, and PGTVrpn.

The CTV included the high-risk CTV (CTVnx and CTVna) and low-risk CTV according to the orderly stepwise pattern of tumor spread and the risk region potentially involved around the nasopharyngeal cavity. For stage T1-2, CTVnx was defined as GTVnx plus a 5-7-mm margin encompassing the entire nasopharyngeal mucosa plus 5 mm of the submucosal volume. For stage T3-4, CTVnx was defined as GTVnx plus a 5-7-mm margin encompassing the entire nasopharyngeal mucosa plus 5 mm of the submucosal volume, parapharyngeal space, pterygoid fossae, and foramen lacerum. PTVnx was created automatically by adding a 1-3-mm margin in three dimensions. For stage N0, CTVna was defined as lymphatic drainage clearance in level II of the bilateral neck, while for stage N1-3, CTVna included GTVnd plus a 2-5-mm margin, the ipsilateral lymph drainage space 2 cm below the GTVnd, and the contralateral lymphatic drainage clearance in level II. PTVna was created automatically by adding a 1-3-mm margin. The low-risk CTV included the CTVnx plus a 5-10 mm margin, CTVnx plus a 2-5-mm margin, and the prophylactic low-risk neck irradiation area. The low-risk PTV was defined as the low-risk CTV plus a 3-mm margin.

**Figure 2:**
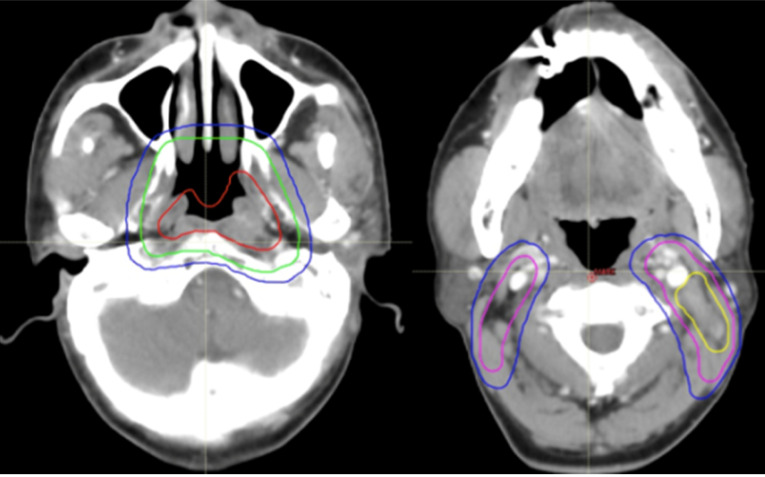
Selected computed tomography slices to demonstrate the delineation of different risk target volumes. Red: PGTVnx; yellow: PGTVnd; green: PTVnx; pink: PTVna; blue: PTV1. Abbreviations: PGTVnx: planning target volume of nasopharyngeal GTV; PGTVnd: planning target volume of GTV in cervical lymph nodes; PTVnx: planning target volume of nasopharynx; PTVna: planning target volune of neck area; PTV: planning target volume 1.

**Figure 3:**
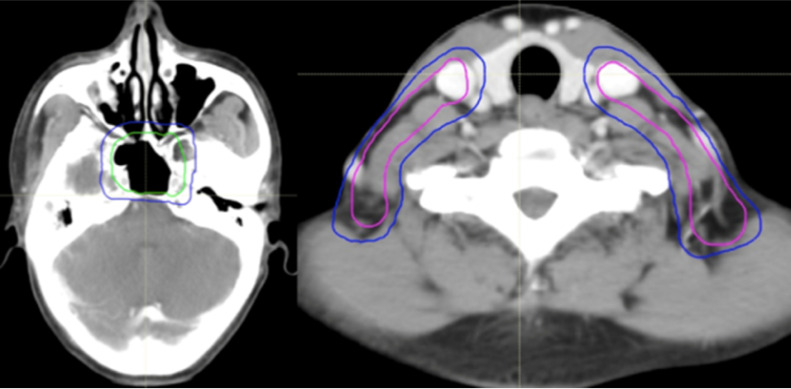
Selected computed tomography slices to demonstrate the delineation of different risk target volumes. Red: PGTVnx; yellow: PGTVnd; green: PTVnx; pink: PTVna; blue: PTV1. Abbreviations: PGTVnx: planning target volume of nasopharyngeal GTV; PGTVnd: planning target volume of GTV in cervical lymph nodes; PTVnx: planning target volume of nasopharynx; PTVna: planning target volune of neck area; PTV: planning target volume 1.

**Figure 4:**
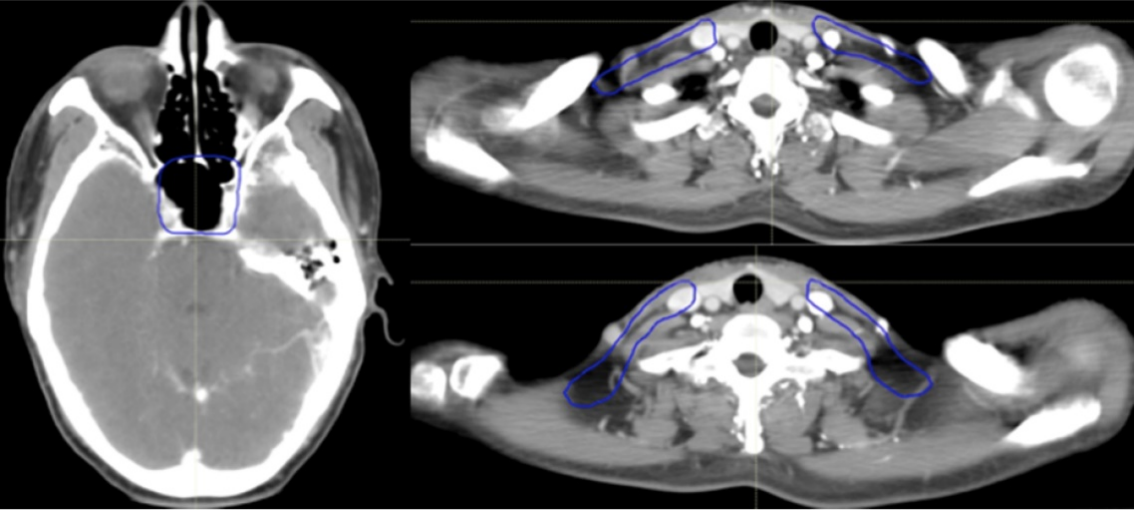
Selected computed tomography slices to demonstrate the delineation of low-risk target volumes. Red: PGTVnx; yellow: PGTVnd; green: PTVnx; pink: PTVna; blue: PTV1. Abbreviations: PGTVnx: planning target volume of nasopharyngeal GTV; PGTVnd: planning target volume of GTV in cervical lymph nodes; PTVnx: planning target volume of nasopharynx; PTVna: planning target volume of neck area; PTV: planning target volume 1.

All of the PTVs, including PGTVnx, PGTVnd, PTVnx, PTVna, and PTV, were trimmed so as not to be delineated outside of the skin surface. Table 1 and Figures 2-4 demonstrate the delineations of different risk target volumes using our protocol.

### General principles of different risk target volume design

In one CT image slice, if there is a GTV, the peripheral structures of the NPC tumor (primary tumor and cervical lymph nodes) invasion should be divided into three tumor volumes of high-, medium-, and low risk such as the PGTVnx/nd, PTVnx/na, and PTV. Moreover, three prescribed dose levels are given for the corresponding risk target volumes.

In one CT image slice, if there is a high-risk area but no GTV, two risk target areas and two prescription dose levels for medium- and low risk should be designed.

In one CT image slice, if there is neither a GTV nor a high-risk area but it includes a low-risk area, a target area and a prescription dose for low risk should be designed.

### Dose prescription

Doses of 70.5, 70.5, 63, 60, and 51 Gy in 30 fractions were administered to the PGTVnx, PGTVnd, PTVnx, PTVna, and PTV, respectively. If PGTVrpn was added into PGTVnx, a dose of 73.5 Gy in 30 fractions was delivered. SIB IMRT was performed once daily, in five fractions per week, over 6 weeks according to the IMRT planning.

### Delineation of organs at risk

Critical normal structures, including the brainstem, spinal cord, parotid glands, optic nerves, chiasm, lens, eyeballs, temporal lobes, temporomandibular joints, mandible, and hypophysis, were contoured and set as organs at risk (OARs) during optimization.

### Chemotherapy

Out of 335 patients, 302 received three-weekly platinum-based IC, 313 underwent three-weekly concurrent chemotherapy with cisplatin, and 199 received adjuvant chemotherapy (AC). The available IC regimens included TPF (docetaxel 60 mg/m^2^/day on day 1, cisplatin 25 mg/m^2^/day on days 1-3, and 5-fluorouracil 500 mg/m^2^/day on days 1-3), TP (docetaxel 60 mg/m^2^/day on day 1, cisplatin 25 mg/m^2^/day on days 1-3), GP (gemcitabine 1,000 mg/m^2^/day on days 1 and 8, cisplatin 25 mg/m^2^/day on days 1-3), and FP (cisplatin 25 mg/m^2^/day on days 1-3, 5-fluorouracil 500 mg/m^2^/day on days 1-3). The one to two cycles of AC consisted of cisplatin and 5-fluorouracil.

### Plan evaluation

Plans were compared by target coverage according to the cross-section dose distribution and the dose-volume histogram (DVH) of the targets. Parameters of the DVH were evaluated as follows: 1) doses received by 95% and 90% of the volumes of the PTV (D95 and D90, respectively), maximum PTV dose (Dmax), minimum PTV dose (Dmin), and mean PTV dose; 2) maximum OARs dose and the volume of OARs receiving a high dose; and 3) the volume received 30 or 40 Gy of the normal tissues.

### Adverse events and survival evaluation

Adverse events were assessed according to the common toxicity criteria of the National Cancer Institute. Survival was calculated from the date of diagnosis to the date of the most recent follow-up, recurrence, or death. The pattern of failure was defined according to the first site of failure: recurrence of the primary tumor or metastasis to regional lymph nodes was regarded as locoregional failure, and metastasis to any site beyond the primary tumor and regional lymph nodes was defined as distant failure.

### Endpoints and statistical analysis

The endpoints of the present study were LRRFS (time from the date of confirmed NPC to locoregional failure), DMFS (time from the date of confirmed NPC to distant metastasis), progression-free survival (PFS) (time from the date of confirmed NPC to progression), OS (time from the date of confirmed NPC to the last follow-up), and acute adverse events from IC and IMRT. If patients relapsed or developed metastasis, they underwent salvage therapy as determined by their physicians. The Kaplan-Meier method was used to generate LRRFS, DMFS, PFS, and OS curves. All data were analyzed using SPSS Statistics version 22.0 (IBM Corp, Armonk, NY).

## Results

### Patient characteristics

From May 2008 to April 2014, the clinical data of 332 untreated, newly diagnosed NPC patients who were initially treated with additional IC followed by SIB IMRT in the Department of Radiation Oncology, Zhejiang Cancer Hospital (Hangzhou, People's Republic of China) were collected and retrospectively reviewed. The basic characteristics of the patients are summarized in Table 2. All patients completed a full course of radical IMRT and received one to four cycles of IC.

### Delineation of different risk target volumes and IMRT planning

**Figure 5:**
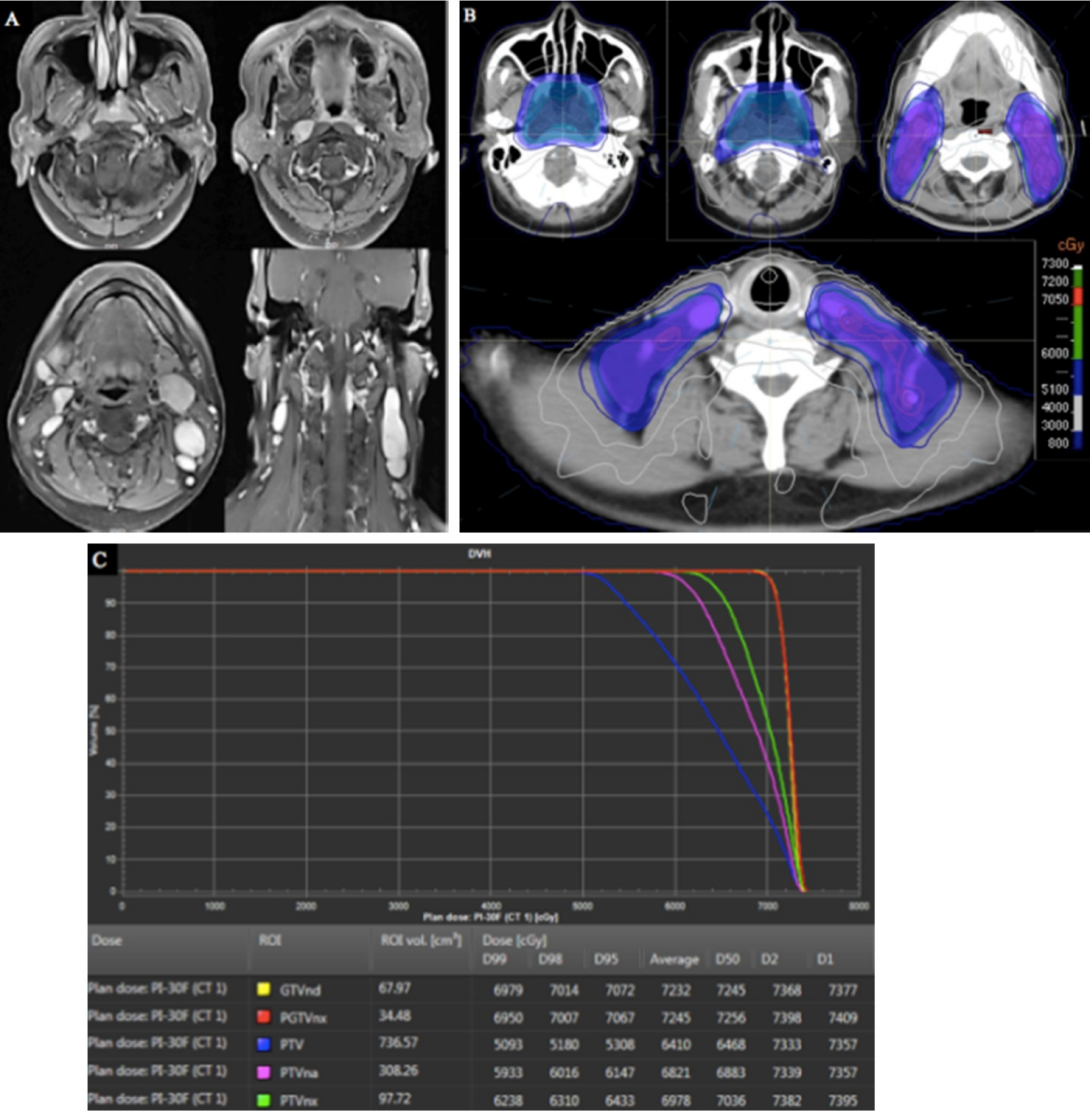
Target volumes and IMRT planning in a patient with stage T2N3M0 NPC. (**A**) MRI of the patient; (**B**) different risk target volumes and dose distributions; (**C**) DVH of IMRT. Abbreviations: IMRT: intensity-modulated radiotherapy; NPC: nasopharyngeal cancer; MRI, magnetic resonance imaging; DVH: dose-volume histogram.

To illustrate our protocol of different risk target volumes, a histology-proven NPC patient was used to describe the different risk target volumes and the corresponding doses in IMRT planning. The MRI of this patient indicated that the primary tumor had invaded the parapharyngeal space, bilateral metastatic RLNs had occurred, and multiple metastatic lymph nodes were observed in the bilateral cervical and supraclavicular regions (Figure 5A). Therefore, the stage was T2N3M0. The patient received three cycles of TP-based IC. After CT simulation following the third IC, we delineated three risk target volumes including high-risk PGTVnx and GTVnd, intermediate-risk PTVnx and PTVna, and low-risk PTV. The IMRT planning was designed by an experienced physicist. Dose distribution was even (Figure 5B). The DVH showed that the prescribed doses met our requirements (Figure 5C).

### Patterns of failure

Among all patients, 47 experienced treatment failure. Twenty-one had locoregional recurrence within the high-dose GTV. Twelve patients developed regional relapse only, and 10 received salvage surgery; eight patients experienced local recurrence only, and one had both local and regional relapse. Twenty-two patients experienced ≥1 distant metastasis, 21 of whom died from disease progression. Four patients experienced locoregional relapse and distant metastasis. Regarding the metastatic location, six patients developed pulmonary metastasis only, eight experienced bone metastases, two developed hepatic metastasis and six developed multiple organ metastases.

### Long-term survival

A total of 407 patients were treated with SIB IMRT. Among them, 36 were excluded from further analysis due to loss to follow-up; thus, 335 participants remained. During the follow-up duration of 70 months, the rates of 3-, 5-, and 7-year LRRFS were 94.1%, 92.3%, and 91.3%, respectively (Figure 7A). The distant metastases-free survival rates at 3-, 5-, and 7-year were 92.7%, 92.1%, and 92.1%, respectively (Figure 7B). The 3-, 5-, and 7-year progression-free survival rates were 88.2%, 85.9%, and 85.0%, respectively (Figure 7C). The rates of OS at 3-, 5-, and 7-year were 90.1%, 86.3%, 82.3%, respectively (Figure 7D). Furthermore, there were no significant differences in LRRFS between T-stage (Figure 8A) and N-stage (Figure 8B) NPC patients.

### Adverse events

For patients treated with IC or AC, adverse events were recorded as IC- or AC-related acute toxicities, while for patients who received RT with or without concurrent chemotherapy (CC), adverse events were recorded as RT-related acute toxicities. If adverse events occurred after a follow-up duration of 6 months, they were recorded as late toxicities. No patient in this study died from treatment-related adverse events.

Table 3 lists the profiles of IC- and RT-related complications. During the period of IC, 94 (28.1%) and 109 (32.5%) patients experienced grade 3-4 leukopenia and neutropenia, 12 and 13 patients developed grade 3-4 anemia and thrombocytopenia, while the rates of grade 3-4 mucositis, diarrhea, and nausea were 2.1%, 2.1%, and 7.5%, respectively. During the period of IMRT, the incidences of grade 3-4 leukopenia, neutropenia, anemia, and thrombocytopenia were 10.4%, 10.1%, 1.5%, and 2.1%, respectively. Moreover, 23 and 11 patients experienced grade 3 mucositis and dermatitis, respectively.

Complications such as dry mouth, hearing loss, carotid stenosis, cranial nerve paralysis, and brain necrosis were regarded as common late adverse events. Of these late adverse events, xerostomia was the most common late complication, and most survivors at the last follow-up experienced mild-to-moderate xerostomia. No grade 2 or higher xerostomia occurred in survivors. However, grade 3 unilateral or bilateral hearing loss was reported in 43 patients (12.8%). Five patients developed cranial nerve damage in the posterior group. Carotid stenosis was observed in two patients. Based on MRI, radiation encephalopathy occurred in 16 patients, two of which developed temporal lobe necrosis.

### Biological effective dose

In our protocol, the high-risk PGTVnx and PGTVnd received doses of 70.5-72 Gy and 69-70.5 Gy, respectively; the intermediate-risk PTVnx and PTVna received doses of 63-66 Gy and 60-63 Gy, respectively; and the low-risk PTV received 51-54 Gy. All doses were administrated in 30 fractions. The biological effective doses (BEDs) of our protocol were calculated using the linear-quadratic model according to the early (α/β = 10) and late (α/β = 3) tissue response parameters. The BEDs of our protocol were similar to the IMRT dose regimens in the China protocol 28 and 0615 protocol 29 (Table 4). Furthermore, after the equivalent formula conversion, the corresponding equivalent doses in 2 Gy/fraction (EQDs2) of our protocol were 73.44, 63.53, 60, and 49.73 Gy in 70.5Gy/30F, 63Gy/30F, 60Gy/30F and 51Gy/30F, respectively.

### Plan comparison

**Figure 6:**
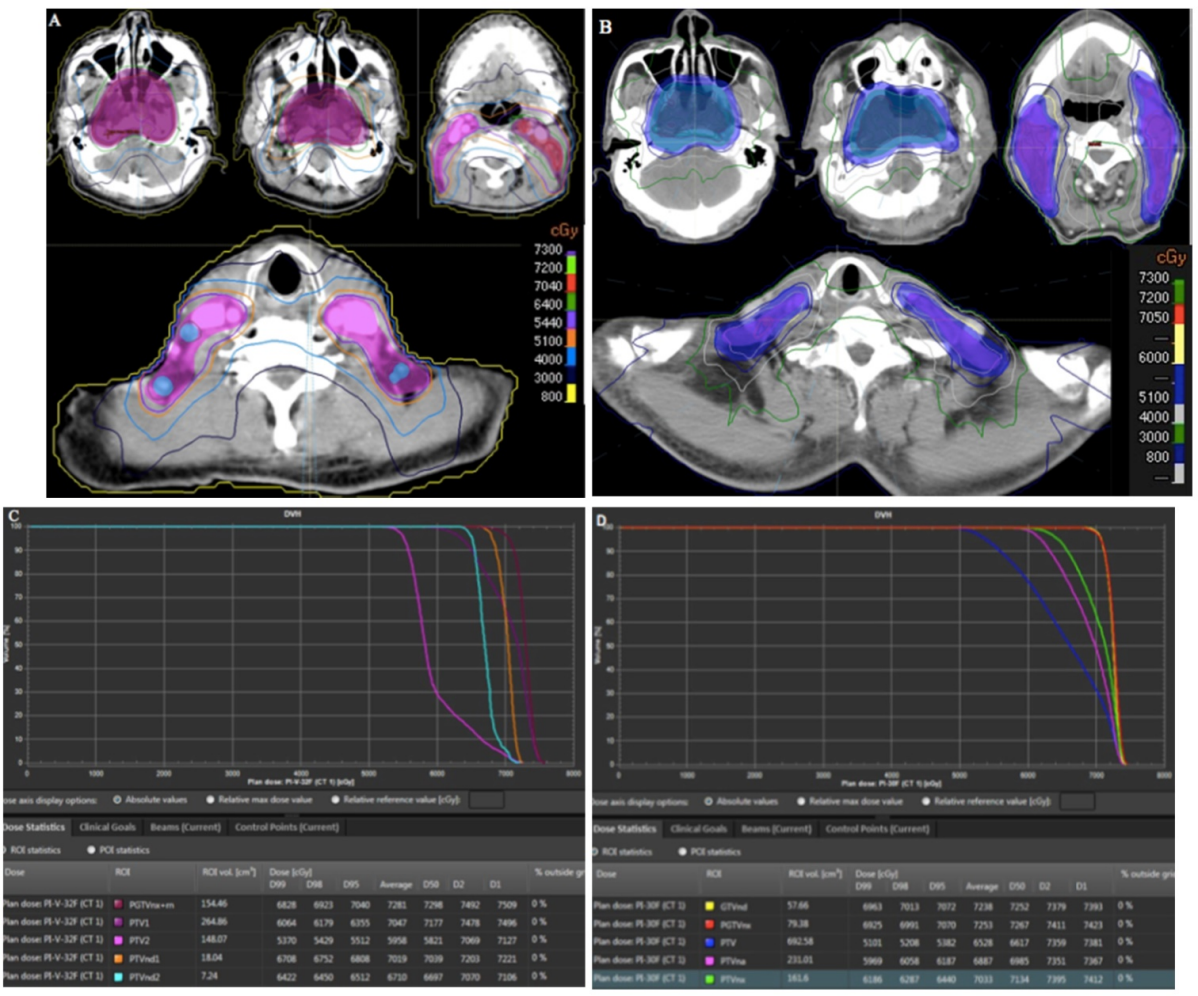
Comparison of target volumes and dose distributions between two protocols. (**A**) target volumes and dose distributions of the China protocol; (**B**) target volumes and dose distributions of our protocol; (**C**) DVH of the China protocol; (**D**) DVH of our protocol. Abbreviation: DVH: dose-volume histogram.

**Figure 7:**
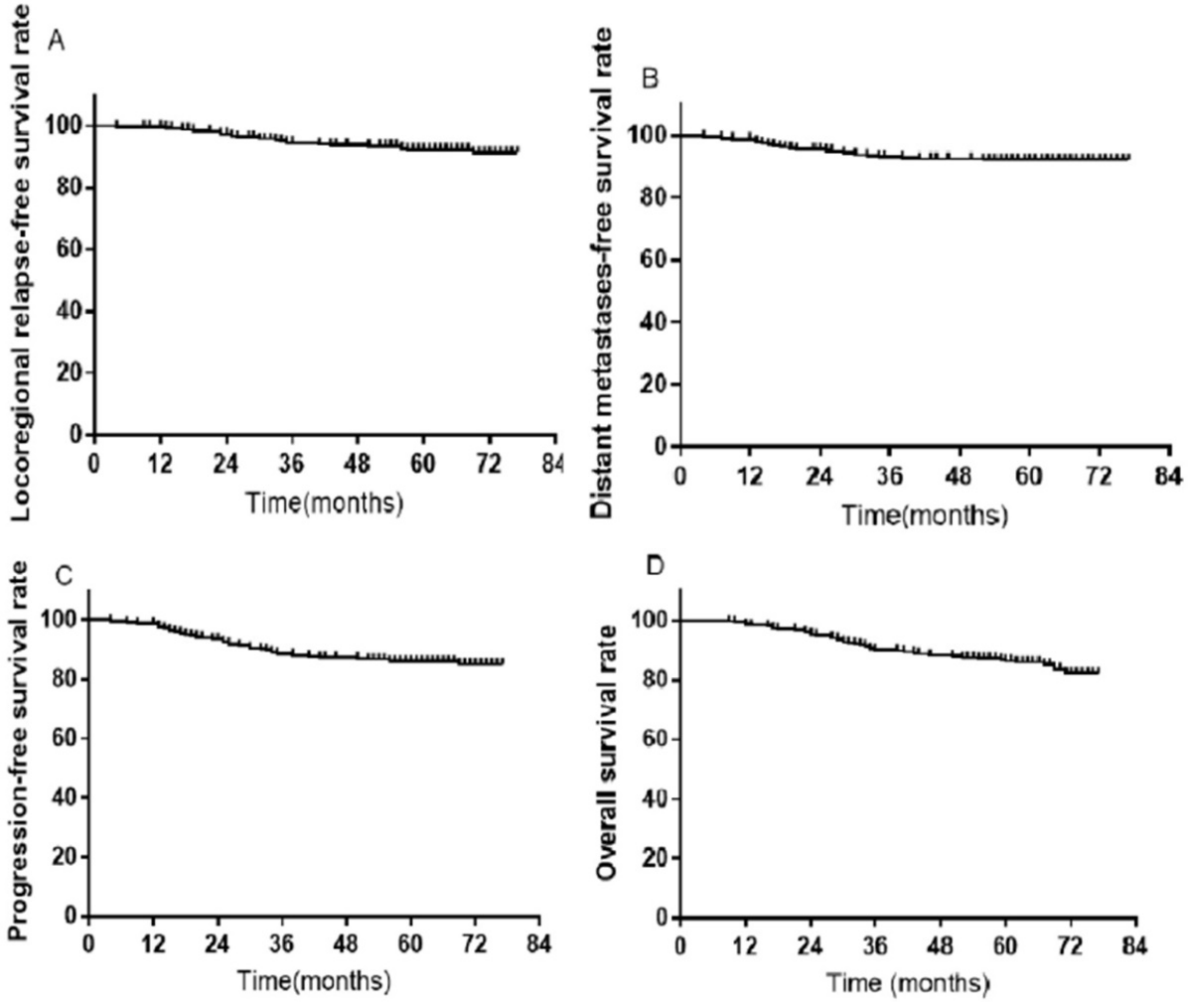
Kaplan-Meier survival curves in 335 NPC patients. (**A**) LRRFS; (**B**) DMFS; (**C**) PFS; (**D**) OS. Abbreviations: NPC: nasopharyngeal cancer; LRRFS: locoregional relapse-free survival; DMFS: distant metastasis-free survival; PFS: progression-free survival; OS: overall survival.

**Figure 8:**
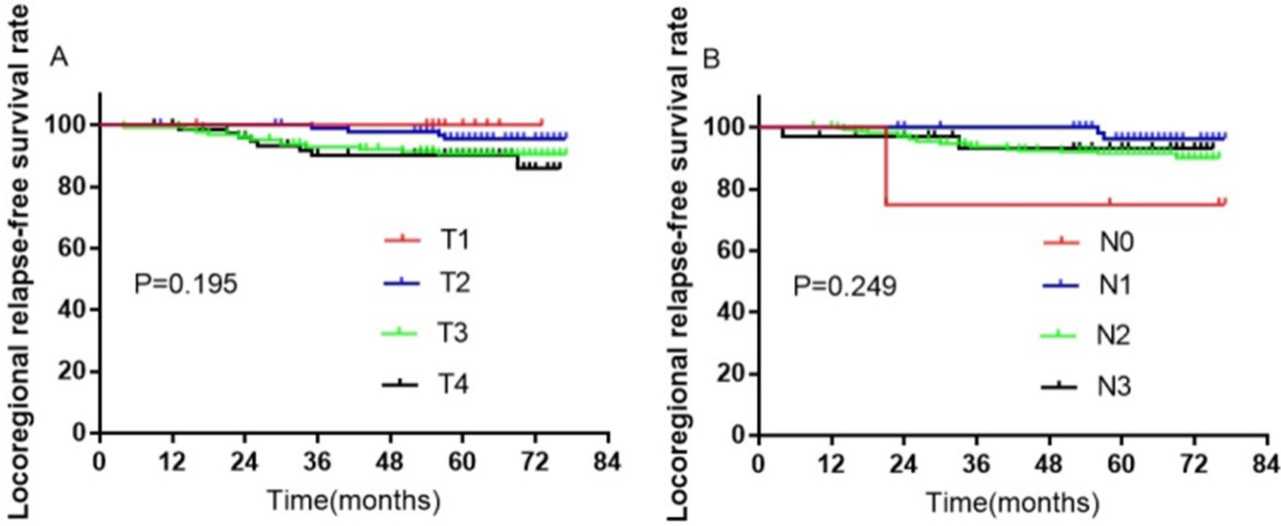
Kaplan-Meier curves of LRRFS in 335 NPC patients with T- or N-stage. (A) T-stage; (B) N-stage. Abbreviations: LRRFS: locoregional relapse-free survival; NPC: nasopharyngeal cancer.

To evaluate the dosimetric benefits of our protocol, we selected two patients with T4-stage NPC. Target volumes for one patient were delineated using the China protocol, and our protocol was used for the other patient. In the China protocol, doses of 70.4, 70.4, 64, 60.8, and 54.4 Gy in 32 fractions were administered to PGTVnx+rn, PGTVnd1, PGTVns2, PTV1, and PTV2, respectively, while doses of 70.5, 70.5, 63, 60, and 51 Gy in 30 fractions were administered to PGTVnx, PGTVnd, PTVnx, PTVna, and PTV. The two IMRT plans met the requirements. Compared with the China protocol, our strategy led to reductions in the V40 and V30 (Figure 5A and 5B) and D2 and D1 (Figure 6C and Figure 6D). For the three risk target volumes, the high dose was more concentrated in the GTV, and the area receiving a low dose was decreased.

## Discussion

According to the 2019 National Comprehensive Cancer Network guidelines, RT plus concurrent chemotherapy with IC or AC is the preferred treatment for NPC. IMRT has shown remarkable promise in the treatment of NPC. However, optimal target volumes and prescribed doses in IMRT for NPC were unclear. A better understanding of the pathway of NPC tumor spread was used to delineate target tumors and increase survival. Here, we described our protocol of different risk target volumes based on the orderly stepwise pattern of tumor spread. Moreover, we report the long-term survival and adverse events of our protocol. The BEDs of the prescribed doses in our protocol were similar to those of the China and 0615 protocols. Further, our protocol resulted in decreases in D1 and D2 in the GTV, while V30 and V40 in normal tissues were lower. In addition, our protocol offered acceptable adverse events and favorable long-term survival outcomes with 5-year LRRFS of 92.2%, 5-year DMFS of 92.1%, 5-year PFS of 85.9%, and 5-year OS of 86.3%. Most importantly, no recurrence occurred in the margins or outside of the high-dose area for all survivors with our protocol.

Because of its highly infiltrative nature, NPC tumors easily invade areas of loose tissue and spread along the lacunae and neural foramen. The extent of invasion and the route of spreading have been described based on MRI findings 30. According to the risk ratios of the invaded anatomic structures around the nasopharynx, Liang et al. 31 and Li et al. 32 divided these anatomic sites into high- (≥50%), medium- (5-30%), and low-risk (<5%) regions. Based on the above results, many Chinese experts with extensive experience in the treatment of NPC proposed a protocol in 2010 28. This protocol is used by most hospitals in China, and favorable survival outcomes have been demonstrated for this protocol. However, it remains unclear whether all the high-risk areas are invaded in each NPC patient. If we include all the high-risk regions and the extension expands uniformly from the GTV, normal tissues are exposed to excessively high doses of radiation. To decrease the doses to normal tissues, reduced target volumes were applied for NPC 15-17, 33. Lin and colleagues 15, 16 used reduced target volumes in IMRT for NPC, and their protocol provided promising local control and survival with acceptable adverse events. Sanford and colleagues 17 designed individual delineations of the CTV for 73 NPC patients treated with IMRT; they found that the 5-year local control, regional control, and OS rates were 94%, 99%, and 84%, respectively. The reduced-volume protocol designed by Billan et al. 33 decreased the volumes of PTV of primary tumor (PTV-P) by 27.6% compared to those in RTOG 0615, and the 3-year disease-free survival was 75%. Given the present situation, international guidelines introduced the principle of the “5 + 5 mm expansion” margin from the GTV (Table 4) 27. This protocol was based on consensus from international experts with experience in the treatment of NPC. The theoretical basis was derived from the data on the microscopic extension from recurrent NPC tumors 34 and other head-neck cancers 35,36. Unfortunately, no clinical survival outcomes using this protocol were reported.

In addition, a better understanding of prescribed doses is needed. In the era of conventional RT, the isocenter dose was often 66-70 Gy in 30 fractions. Chau et al. 37,38 used a three-dimensional treatment planning system to evaluate the actual dosimetric distribution of Ho's technique for NPC; the results showed that the D95s of the GTV and PTV in an isocenter dose of 66 Gy are about 57 Gy and 45 Gy, respectively. Kam et al. 39 indicated that Ho's technique offers a D95 of 62.5 Gy in the GTV and 52.5 Gy in the PTV for T1 NPC patients, a D95 of 63 Gy in GTV and 57.5 Gy in PTV for T2 patients, and a D95 of 65 Gy in GTV and 55 Gy in PTV for T4 cases. Our previous study demonstrated that conventional RT techniques provide 63.8 Gy for D95 in PGTVnx, 63 Gy for D95 in PTVnx, and 42.5 Gy for D95 in PTV 40. Although the dose distributions of conventional RT techniques were unsatisfactory, the local control rate was about 80%. Based on these results, the prescribed doses of IMRT were 66-70 Gy to the GTV. However, an insufficient dose to the GTV in T3-4 NPC patients treated with IMRT was related to poor local control 41. Thus, data of appropriate prescribed doses for target volumes are lacking.

Accordingly, we divided the tumor and the anatomic sites around the tumor into three or four risk target volumes based on the orderly stepwise pattern of tumor spread (Table 1). High-risk PGTVnx and PGTVnd were irradiated with high doses, intermediate-risk PTVnx and PTVna were irradiated with intermediate doses, and low-risk PTV was irradiated with low doses. If the RLNs were large or resistance to IC, we add PGTVrpn to PGTVnx and administered a higher dose. Using our protocol, the high dose was more concentrated in the GTV, and the areas receiving a low dose were decreased. The BEDs of our protocol were similar to those of the IMRT dose regimens in the China and 0615 protocols (Table 4). Moreover, the corresponding EQDs2 of our protocol were 73.44, 63.53, 60, and 49.73 Gy in 70.5Gy/30F, 63Gy/30F, 60Gy/30F and 51Gy/30F, respectively. Notably, the LRRFS, DMFS, PFS, and OS rates at 5 years were 92.2%, 92.1%, 85.9%, and 86.3%, respectively.

Although the present protocol provided favorable dosimetric parameters and survival outcomes with fewer acute and late adverse events, several limitations of our study should be noted. First, as this was a single-center study, all the limitations associated with single centers apply. Second, due to the retrospective nature of the study, it provided relatively low power to indicate the superiority of our protocol. Third, acute adverse events were assessed according to medical records; no quality-of-life assessments were performed for long-term survivors. Moreover, the effect of IC has not been considered in our protocol. Thus, our protocol should be regarded as preliminary. Our revised protocol should be verified in future prospective and large-sample clinical trials.

## Conclusion

We presented a protocol of different risk target volumes and corresponding prescribed doses for IMRT in NPC patients. Our protocol resulted in superior dose distributions for reducing high-dose volumes in the GTV and decreasing low-dose volumes in normal tissues. The present regimen provided favorable long-term survival outcomes with acceptable acute and late adverse events. Therefore, this protocol can be recommended for the treatment of NPC.

### Funding

This study was supported by grants from the Medical and Health Science and Technology Program of Zhejiang Province (No. 2020KY084, No. 2019KY041, No. 2013KYB033, No. 2009B026, No. 2006A016, No. 2005B012, and No. 2004B014) and the National Natural Science Foundation of China (No. 81502647).

## Figures and Tables

**Table 1:** Delineation of different risk target volumes using our protocol

Target volume	Definition
GTV in NP	GTVnx
GTV of LN	GTVnd
High-risk target volumes	PGTVnx = GTVnx + 0-3 mm + whole NP (5 mm submucosal) PGTVnd = GTVnd + 3 mm
Intermediate-risk target volume for primary tumor	T1-2: CTVnx = GTVnx + 5-7 mm T3-4: CTVnx = GTVnx + 5-7 mm + whole NP (5 mm submucosal) + pterygoid + foramen lacerum PTVnx = CTVnx + 1-3 mm
Nasal cavity-posterior part	3 mm from the choana
Maxillary sinuses-posterior part	3 mm from the posterior wall
Posterior ethmoid sinus	If invasion,
Skull base	Cover foramina ovale, rotundum, and lacerum
Cavernous sinus	Cover side involved only if T3-4
Pterygoid fossae	+
Parapharyngeal spaces	Full coverage
Sphenoid sinus	Inferior half if no invasion; whole if invasion
Clivus	Anterior third if no invasion; whole if invasion
Intermediate-risk target volume for cervical lymph node	CTVna: cover bilateral level II plus VA if N0; cover bilateral level II, VA, plus at least one level ipsilateral below the involved levels PTVna = CTVna + 1-3 mm
Bilateral RP, level II, III, IVa	Bilateral level II plus VA if N0; bilateral level II, VA, plus at least one level ipsilateral below the involved levels
Level IB	IB LN+ve IIA LN+ve Invaded structure that drains to level IB as first echelon site
Low-risk target volume	CTV: CTVnx + 3-7 mm for primary site; CTVna + 2-5 mm for cervical LN; cover lower neck and supraclavicular if N1-3 or omit if N0 PTV = CTV + 1-3 mm

GTV: gross tumor volume; NP: nasopharynx; LN: lymph node; CTV: clinical target volume; PTV: planning target volume; GTVnx: GTV of nasopharynx; GTVnd: GTV of cervical lymph nodes; PGTVnx: planning target volume of nasopharyngeal GTV; PGTVnd: planning target volume of GTV in cervical lymph nodes; CTVnx: CTV of nasopharynx; PTVnx: planning target volume of CTV; CTVna: clinical target volume of neck area.

**Table 2:** Baseline characteristics of the 335 newly diagnosed NPC patients enrolled in the study

Characteristic	Number of patients	%
**Age at diagnosis**		
Range (years)	17-79	
Median age (years)	50	
**Sex**		
Male	240	71.6
Female	95	28.4
T stage^*^		
T1	13	3.9
T2	101	30.1
T3	143	42.7
T4	78	23.3
N stage^*^		
N0	4	1.2
N1	64	19.1
N2	235	70.1
N3	32	9.6
Clinical stage^*^		
II	26	7.8
III	208	62.1
IV	101	30.1
**IC regimens**		
TPF	54	16.1
TP	155	46.3
GP	71	21.2
PF	22	6.6
No	33	9.8
**Treatment modality**		
IC+CRT+AC	165	49.3
IC+IMRT+AC	14	4.2
IC+CRT	115	34.3
IC+IMRT	8	2.4
CRT	13	3.9
CRT+AC	20	5.9

IC: induction chemotherapy; CRT: concurrent chemoradiotherapy; AC: adjuvant chemotherapy; IMRT: intensity-modulated radiotherapy; TPF: docetaxel/cisplatin/fluorouracil; TP: docetaxel/cisplatin; GP: gemcitabine/fluorouracil; FP: cisplatin/fluorouracil. *American Joint Committee on Cancer/International Union against Cancer staging system, seventh edition.

**Table 3:** Profile of IC- and RT-related acute toxicities

Adverse events	During IC (n)				During IMRT (n)			
	1	2	3	4	1	2	3	4
**Hematologic**								
Leukopenia	35	92	58	36	76	53	32	3
Neutropenia	41	62	57	52	68	47	29	5
Anemia	93	29	11	1	42	17	5	0
Thrombocytopenia	55	18	8	5	33	13	5	2
Liver function	36	15	6	0	10	5	0	0
Renal function	9	2	1	0	0	0	0	0
**Non-hematologic**								
Mucositis	25	16	6	1	181	123	23	0
Dermatitis	37	5	0	0	261	57	11	0
Diarrhea	31	19	7	0	13	2	0	0
Nausea/vomiting	82	58	23	2	35	22	13	0

IC: induction chemotherapy; RT: radiotherapy; IMRT: intensity-modulated radiotherapy.

**Table 4:** Comparison of biological equivalent doses among three protocols

Variable	Present protocol		China protocol		0615/0225 protocol	
α/β	Dose	BED	Dose	BED	Dose	BED
3	70.5 Gy/30F	125.73	70.4 Gy/32F	122.03	70 Gy/33F	119.40
10		88.13		85.89		84.79
3	63 Gy/30F	107.1	64 Gy/32F	106.67	62.7 Gy/33F	102.41
10		76.23		85.89		74.61
3	60 Gy/30F	100	60.8 Gy/32F	99.31	59.4 Gy/33F	95.04
10		72		72.35		70.1
3	51 Gy/30F	79.9	54.4 Gy/32F	85.23	50.4 Gy/33F	80.64
10		59.67		63.65		59.47

## Abbreviations

AC: adjuvant chemotherapy
BED: biological effective dose
CT: computed tomography
CTV: clinical target volume
CTVna: CTV of neck area
CTVnx: CTV of nasopharynx
DMFS: distant metastasis-free survival
DVH: dose-volume histogram
EQDs2: equivalent doses in 2 Gy/fraction
GTV: gross tumor volume
GTVnd: GTV of cervical lymph nodes
GTVnx: GTV of nasopharynx
GTVrpn: GTV of retropharyngeal lymph nodes
IC: induction chemotherapy
IMRT: intensity-modulated radiotherapy
LRRFS: locoregional relapse-free survival
MRI: magnetic resonance imaging
NPC: nasopharyngeal cancer
OAR: organ at risk
OS: overall survival
PFS: progression-free survival
PGTVnd: planning target volume of GTV in cervical lymph nodes
PGTVnx: planning target volume of GTV in nasopharynx
PGTVrpn: planning target volume of GTV in retropharyngeal lymph nodes
PTV: planning target volume
PTVna: PTV of neck area
PTVnx: PTV of nasopharynx
RLN: retropharyngeal lymph node
RT: radiotherapy
SEER: Surveillance, Epidemiology, and End Results
SEQ: sequential
SIB: simultaneously integrated boost
V30: the volume received 30
V40: the volume received 40 Gy
